# 52. Direct Communication Improves Response Time to Acceptance of Antimicrobial Stewardship Interventions

**DOI:** 10.1093/ofid/ofab466.254

**Published:** 2021-12-04

**Authors:** Humaira Shafi, Stephen G Donoghue, Jonathan Seah, Pu En Ow Yong, Wee Boon Lee

**Affiliations:** 1 Changi General Hospital, Singapore, Not Applicable, Singapore; 2 MPSI, Singapore, Not Applicable, Singapore; 3 Changi General Hospital, Singapore, Singapore, Not Applicable, Singapore

## Abstract

**Background:**

Hospital antimicrobial stewardship program (ASP) reviews broad-spectrum antibiotics and recommends interventions to optimise antimicrobial use. However, about 30% of interventions are not accepted. This project aims to improve the response rate and time for acceptance of ASP interventions by direct communication with providers (via call or text messaging) once an intervention was made.

**Methods:**

Pre-direct communication (PC) phase lasted from 1^st^ Jan - 31^st^ Dec 2017. A typed intervention was placed into the patient’s medical records for the team to review. Thereafter, a direct communication (DC) phase ran from 1^st^ Jan 2018 - 31^st^ Jan 2019. Teams were immediately notified of any ASP interventions made via a call or text message, in addition to the document placed in the medical records. Specialty, acceptance rates, type of intervention and time to acceptance was recorded. Overall acceptance was counted if team followed the ASP recommendations within 48 hours.

**Results:**

A total of 621 interventions were made over the 25-month period (PC n=334, DC n=287). We found that direct communications did not improve the overall acceptance rates (PC 66% vs. DC 65%, p=0.791), but significantly improved same day acceptance rates (PC 15% [49/334] vs. DC 33% [96/287], p< 0.001). This trend for higher same-day acceptance was also noted regardless of specialty. It increased from 15% to 45% (p< 0.001) for medicine & 15% to 25% (p=0.025) for surgery. Furthermore, overall acceptance for medical discipline was significantly higher in the DC phase (68% to 80%, p=0.024); no significant difference noted for the surgical disciplines. Same-day acceptance also improved when we compared the most common types of interventions (culture based de-escalation, discontinue antibiotic, narrow empirical coverage). In addition, DC helped narrow empiric antibiotic choices, with improvements in both same-day and overall acceptance of interventions (increased from 8% to 43%, p< 0.001 and 57% to 78%, p=0.12, respectively).

**Conclusion:**

Direct communication with clinicians boosted same-day acceptance for ASP interventions. In addition, it increased overall acceptance for medical disciplines, and to narrow empiric antibiotic use. Future efforts will focus on in-person strategy with surgical teams for fruitful results.

**Disclosures:**

**All Authors**: No reported disclosures

